# Extracellular Vesicles in Human Reproduction: Integrating Redox–Mitochondrial Signaling with Multi-Omics and AI-Driven Biomarker Discovery

**DOI:** 10.3390/cells15100955

**Published:** 2026-05-21

**Authors:** Sofoklis Stavros, Angeliki Gerede, Efthalia Moustakli, Athanasios Zikopoulos, Ioannis Tsakiridis, Christina Messini, Anastasios Potiris, Ismini Anagnostaki, Ioannis Arkoulis, Spyridon Topis, Themistoklis Dagklis, Dimitrios Loutradis

**Affiliations:** 1 Third Department of Obstetrics and Gynecology, University General Hospital “ATTIKON”, Medical School, National and Kapodistrian University of Athens, 12462 Athens, Greece; apotiris@med.uoa.gr (A.P.); spyros.topis1996@gmail.com (S.T.); 2 Unit of Maternal-Fetal-Medicine, Department of Obstetrics and Gynecology, Medical School, Democritus University of Thrace, 68100 Alexandroupolis, Greece; agerede@otenet.gr; 3 Department of Nursing, School of Health Sciences, University of Ioannina, 45500 Ioannina, Greece; ef.moustakli@uoi.gr; 4 Obstetrics and Gynecology, Royal Cornwall Hospital, Truro TR1 3LJ, UK; thanzik92@gmail.com; 5 Third Department of Obstetrics and Gynecology, General Hospital Ippokratio, Medical School, Aristotle University of Thessaloniki, 54642 Thessaloniki, Greece; iotsakir@gmail.com (I.T.); tdagklis@gmail.com (T.D.); 6 Department of Obstetrics and Gynaecology, Faculty of Medicine, School of Health Sciences, University of Thessaly, 41500 Larisa, Greece; messini.uth@gmail.com; 7 Medical School, National and Kapodistrian University of Athens, 11528 Athens, Greece; isanagnostaki3@gmail.com; 8 First Department of Obstetrics and Gynecology, Alexandra Hospital, Medical School, National and Kapodistrian University of Athens, 11528 Athens, Greece; garkoylis@hotmail.com; 9 Fertility Institute-Assisted Reproduction Unit, Paster 15, 11528 Athens, Greece; loutradi@otenet.gr

**Keywords:** exosomes, oxidative stress, mitochondrial dysfunction, ART, embryo implantation, endometrial receptivity, biomarker discovery, precision medicine

## Abstract

In the human reproductive system, extracellular vesicles (EVs) have been recognized as playing a vital role in mediating cell–cell communication. They are considered critical for embryo development, implantation, gamete interaction, and fertilization. The various cargoes carried by EVs, depending on the physiological and pathological state of the cell, include proteins, lipids, nucleic acids, and mitochondrial components. EVs are recognized as critical carriers of redox-related signals and mitochondrial components, linking oxidative stress (OS) to reproductive failure and influencing gamete quality and embryo competence. Although considerable progress has been made, research remains poorly integrated, despite individual omics technologies providing valuable molecular insights. The use of multi-omics technologies, including transcriptomics, proteomics, metabolomics, and microbiome analysis, has been proposed as a global approach to understanding the complexities associated with EVs and discovering new biomarkers associated with infertility. ML and AI have been proposed to identify predictive signatures linked to ART effectiveness and reproductive outcomes, with a strong capacity to handle high-dimensional data. The review aims to provide an overview of current knowledge on EV-mediated redox–mitochondrial signaling in human reproduction, while highlighting the importance of emerging multi-omics and AI technologies for EV-mediated biomarker development. The review discusses the promise of EVs in the development of minimally invasive diagnostic approaches and therapeutic interventions, as well as the challenges in the standardization, integration, and clinical translation of EV-mediated research. In addition, the review proposes integrating computational approaches to better understand molecular pathways involved in the development of next-generation precision medicine in human reproduction.

## 1. Introduction

The issue of infertility has become a major public health problem, affecting a large percentage of couples worldwide, with multifactorial etiologies involving genetic, environmental, metabolic, and lifestyle factors [1,2]. Despite major advances in assisted reproductive technologies (ART), including intracytoplasmic sperm injection and in vitro fertilization, conception rates remain suboptimal, and reproductive outcomes cannot be accurately predicted [3,4]. The molecular mechanisms that govern gamete quality, fertilization, embryo development, and implantation involve complex interactions that cannot be accounted for by current diagnostic tools, many of which involve invasive procedures. Clearly, there is an urgent need for innovative, non-invasive diagnostics and molecular understanding, which will help stratify patients and personalize reproductive therapy [5,6,7].

In both physiological and pathological conditions, extracellular vesicles (EVs) have emerged as critical regulators of cell–cell interactions [8]. Exosomes, microvesicles, and apoptotic bodies are membrane-bound structures that are secreted by most cell types and found in various biological fluids, including semen, follicular fluid (FF), oviductal fluid, uterine secretions, and blood [9,10]. Proteins, lipids, messenger RNA (mRNA), microRNA (miRNA), and other non-coding RNA, along with mitochondrial components, form part of the heterogeneous and biologically active cargo carried by EVs [11]. Most importantly, EVs are potential candidates for non-invasive biomarkers because their composition directly corresponds to the functional status of the cells that release them [12]. The major aspects of human reproduction, including sperm maturation, oocyte competence, fertilization, embryo-maternal communication, and endometrial receptivity, have been associated with EVs [13].

The role of oxidative stress (OS) and mitochondrial dysfunction in reproductive success is a new area of focus in the field of reproductive biology [14]. While reactive oxygen species (ROS) are important for cell signaling at physiological levels, excessive production of ROS results in oxidative damage to proteins, lipids, and nucleic acids, ultimately affecting gametes and embryonic development [15,16]. The entire process is mediated through mitochondria, as this is the major power house of the cell, responsible for maintaining the redox balance. Reduced sperm motility, poor oocyte quality, low embryo viability, and failed implantation have all been strongly correlated with defective mitochondrial function [14,17]. EVs mediate intercellular signaling and regulate OS in the reproductive environment by transporting redox-related molecules and mitochondria [18].

Tremendous progress has been made in identifying the diverse molecular constituents present in EV cargo, research in this field remains fragmented due to the widespread use of isolated analytical strategies. Omics approaches, including transcriptomics, proteomics, metabolomics, and lipidomics, enable comprehensive profiling of EV cargo and reveal complex molecular signatures related to reproductive function and disease [19,20]. Nevertheless, single-omics analyses often fail to capture the intricate interactions among molecular layers and may therefore provide only a partial view of underlying biological processes. Multi-omics integration offers a broader systems-level perspective by combining diverse datasets to identify interconnected pathways and more robust biomarkers [21].

Recent advances in machine learning (ML) and artificial intelligence (AI) have further expanded the analytical capabilities for EV research [22]. These technologies are capable of identifying complex, non-linear interactions and are particularly well-suited for analyzing high-dimensional, multi-layered datasets. ML is increasingly being used to evaluate endometrial receptivity, determine the outcomes of ART, and evaluate embryo quality in reproductive medicine [23]. When integrated with EV-derived multi-omics data, AI-driven analyses may facilitate the identification of predictive biomarker signatures and support the development of precision reproductive machine strategies [19].

Despite these advances, several barriers continue to limit the clinical translation of EV-based biomarkers. The challenges are varied and include those related to sample sources, reproducibility, integration of heterogeneous datasets, and the lack of standardized methods for isolating and characterizing EVs [24]. Furthermore, rigorous validation studies remain necessary to ensure the reliability and generalizability of proposed biomarkers across diverse patient populations [25].

**Figure 1:**
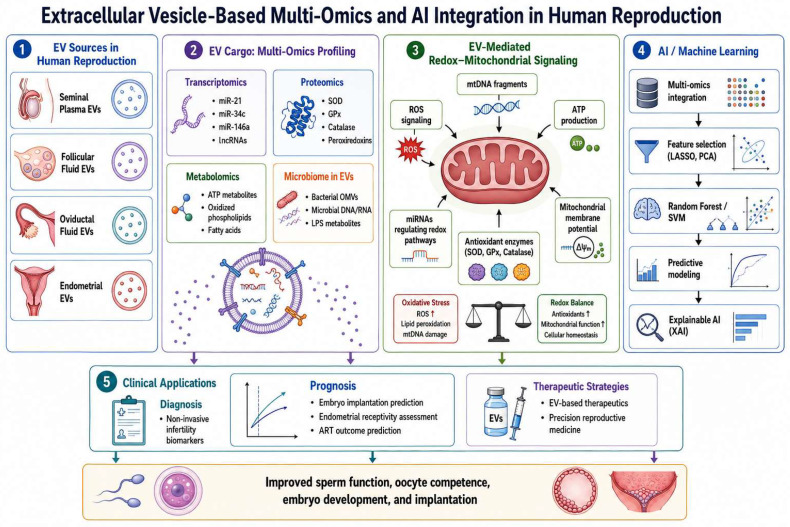
Integrated conceptual framework of EV-mediated multi-omics profiling and AI-driven biomarker discovery in human reproduction. EVs derived from reproductive sources, including seminal plasma, FF, oviductal fluid, and endometrial secretions, carry diverse molecular cargo comprising transcriptomic, proteomic, metabolomic, and microbiome-associated components. These molecular layers converge on redox–mitochondrial signaling pathways, which play a central role in regulating gamete quality, fertilization, embryo development, and implantation. Integration of multi-omics EV data through AI and machine learning approaches enables the identification of predictive biomarkers and supports clinical applications, including diagnosis, prognosis of ART outcomes, and development of targeted therapeutic strategies. The figure illustrates the integrative framework proposed in this review linking EV biology, redox regulation, mitochondrial signaling, multi-omics technologies, and precision reproductive medicine.

Infertility issues, EV biogenesis, reproductive communication, and therapeutic uses have all been the subject of separate reviews of EVs in reproductive biology [10,13,24]. In contrast, the present review suggests an integrated paradigm that connects EV-mediated intercellular communication to mitochondrial signaling, OS management, multi-omics profiling, and AI-assisted biomarker identification in human reproduction [18,19,20,21,22,23]. Particular emphasis is placed on the emerging role of EVs as mediators of redox–mitochondrial crosstalk during gametogenesis, fertilization, embryo development, and endometrial receptivity [18]. Furthermore, this study critically assesses the integration of AI/ML techniques with EV-derived multi-omics datasets, translational difficulties, and existing methodological restrictions. By combining mechanistic, technological, and translational perspectives, this review aims to provide a comprehensive overview of the evolving role of EVs in reproductive biology and assisted reproduction. The proposed integrative framework is illustrated in Figure 1.

## 2. Literature Search Strategy

A narrative literature search was conducted to identify relevant studies examining EVs, OS, mitochondrial signaling, multi-omics technologies, and AI/ML applications in human reproduction and ART. The databases PubMed/MEDLINE, Scopus, and Google Scholar were used for the searches. The literature search primarily included studies published between 2010 and February 2026, with additional seminal earlier studies included where scientifically relevant.

Search terms included combinations of the following keywords: “extracellular vesicles”, “exosomes”, “human reproduction”, “fertility”, “assisted reproductive technologies”, “oxidative stress”, “reactive oxygen species”, “mitochondrial dysfunction”, “multi-omics”, “transcriptomics”, “proteomics”, “metabolomics”, “artificial intelligence”, “machine learning”, and “biomarkers”.

Translational research articles, original studies, clinical investigations, experimental studies, and pertinent review papers published in English were all deemed appropriate for inclusion. Excluded studies had insufficient methodological information, were unrelated to reproductive biology, or had no bearing on the finding of biomarkers or EV-mediated signaling. Priority was given to recent research, mechanistic investigations, therapeutically relevant data, and studies that combined EV biology with omics technology or AI-driven analytical tools.

The current study was designed as a narrative and integrative evaluation rather than a conventional systematic review or meta-analysis because the area is multidisciplinary and quickly changing. To present a thorough summary of recent mechanistic, technical, and translational developments in EV research within reproductive medicine, the literature synthesis was selective and concept-driven.

## 3. Biology of EVs in Human Reproduction

EVs are a heterogeneous population of membrane-bound particles involved in intracellular communication through the transport of bioactive molecules [12]. EVs are secreted from different cell types in the human reproductive system, including the reproductive fluids in which they are found, i.e., uterine secretions, FFs, oviductal fluids, and seminal plasma [26,27]. EVs are involved in the coordination of complex biological processes, which are all crucial in the success of the reproductive process, including gamete maturation, fertilization, development, and implantation [28]. The ability of EVs to transport proteins, lipids, nucleic acids, and other components of organelles enables them to function as dynamic regulators in the reproductive system [9].

### 3.1. EV Biogenesis and Classification

Based on biogenesis, size, and composition, EVs can be broadly classified into three types: apoptotic bodies, microvesicles, and exosomes. Despite similarities in size and composition, their functional differences arise from distinct biogenesis pathways [29].

Exosomes are formed within the endosomal compartment and typically range from 30 to 150 nm in diameter [29]. Intraluminal vesicles are formed in multivesicular bodies, which are formed due to the inward budding of the plasma membrane of early endosomes [30]. Exosomes are released from the cell when these multivesicular bodies fuse with the plasma membrane. This process is mediated by both ESCRT-dependent and ESCRT-independent pathways involving key proteins such as Alix, TSG101, and tetraspanins (CD9, CD63, and CD81). Exosomes are characterized by the presence of certain proteins and RNAs, which are unique in nature, and are enriched in certain lipids, including cholesterol and sphingomyelin [31,32].

Straight out budding and fission of the plasma membrane result in the formation of microvesicles (100–1000 nm). This process is driven by changes in lipid asymmetry, elevated intracellular calcium levels, and cytoskeletal remodeling [33]. Microvesicles can be indicative of the cell’s immediate responses to environmental stimuli, such as stressful events, and are composed of cytosolic proteins and RNA [34].

Apoptotic bodies (500–5000 nm) are formed during programmed cell death and are composed of cell components like DNA, organelles, and cytoplasmic fragments [35]. Although these structures have traditionally been associated with cell clearance, recent evidence suggests they also play important roles in immune regulation and intercellular communication [36].

It is noteworthy that EV populations are highly heterogeneous, and existing techniques for isolating EVs often yield heterogeneous populations of vesicles [37].

Different EV isolation techniques may substantially influence EV purity, structural integrity, and preservation of redox-associated cargo [24,29]. Ultracentrifugation, although widely used, may induce vesicle aggregation, membrane disruption, oxidative modifications of proteins and lipids, and co-isolation of non-EV contaminants due to prolonged high-speed centrifugation [29]. In contrast, size-exclusion chromatography generally pro-vides improved preservation of EV structural integrity and reduced protein contamination but may yield lower particle concentrations. Emerging microfluidic-based platforms offer rapid processing and increased sensitivity for small sample volumes; however, their reproducibility and standardization remain limited [37]. These methodological differences may significantly affect downstream multi-omics analyses, oxidative stress-associated biomarker detection, and the biological interpretation of EV-mediated signaling pathways [24,37].

In this regard, characterization based on physical characteristics, biochemical markers, and cellular origin is advised by definitions and frameworks such as the MISEV guidelines developed by the International Society for Extracellular Vesicles [38,39]. In reproductive biology, EV biogenesis is strongly influenced by hormonal signals, cell metabolism, and microenvironmental circumstances. These factors facilitate communication between reproductive cells in a highly specific manner through dynamic changes in EV contents [40,41].

### 3.2. EVs in Male Reproduction

EVs play an important role in sperm maturation, functional competence, and fertilizing ability in the male reproductive system [42]. Epididymosomes, secreted by epididymal epithelial cells, are some of the best-studied EVs in this microenvironment. Spermatozoa undergo crucial maturation events during epididymal transit, including acquisition of motility and fertilizing ability [43,44,45]. Spermatozoa acquire proteins, lipids, and regulatory RNA from epididymosomes, leading to changes in membrane composition, signaling, and metabolic activity [46].

A large number of EVs of prostate, seminal vesicle, and other accessory gland origin have been reported in seminal plasma [47]. These EVs supply components necessary for sperm capacitation, motility, and protection against OS. To maintain sperm integrity in the oxidative environment of the male and female reproductive tract, for example, EV-borne enzymes and antioxidants can regulate the redox balance [47].

In the female reproductive system, following ejaculation, the role of EVs is also related to immunological regulation. By promoting tolerance to paternal antigens and creating conditions conducive to conception and embryo development, seminal EVs can influence the mother’s immune system [48,49].

The alteration of EV components is linked to male infertility. The miRNAs, proteins, and OS markers present in EVs are changed in asthenozoospermia and oligozoospermia. EV profiling may provide valuable information on sperm dysfunction and serve as a source of a non-invasive biomarker [47].

### 3.3. EVs in Female Reproduction

EVs play a similar role in folliculogenesis, oocyte maturation, fertilization, and embryo implantation in the female reproductive system. EVs of granulosa cells, theca cells, and other follicular structures are present in the FF surrounding the developing oocyte. EVs are known to play a significant role in the interaction between the oocyte and somatic cells [50,51].

EVs of the epithelial cells of the oviduct are significant in controlling the activity of sperm and conception. In addition to the essential components required to promote the development of the embryo, EVs of the oviduct can regulate the capacitation of sperm, the acrosome reaction of sperm, and the movement of sperm. EVs contribute to oocyte fertilization by creating a microenvironment that resembles physiological conditions [13,49,52].

Endometrial EVs are crucial for the communication that exists between the mother and the embryo during the time of implantation. The immune system, trophoblast invasion, and endometrial receptivity are also influenced by endometrial EVs. The signaling molecules that are crucial for the success and timing of implantation are exchanged through endometrial and embryo-derived EVs. The disturbances that are experienced during pregnancy and implantation have been attributed to the regulation of these systems [53,54,55].

The role that EVs play in relation to the immune system and microbiome of the reproductive tract was also noted in recent studies. The utilization of EV signaling guarantees that a balanced environment is maintained for a successful reproduction outcome, despite the fact that disruptions may result in endometriosis and implantation failure. The role that EVs play is crucial to the female reproductive system, ensuring that intricate signaling is maintained for a successful conception outcome. The molecular specificity and availability of these vesicles make them significant for use as a diagnostic and prognostic tool [41,56]. The diverse roles of EVs across male and female reproductive systems are summarized in Table 1.

## 4. EV-Mediated Redox–Mitochondrial Signaling

The concept of EVs having a crucial role in facilitating redox and mitochondrial signaling within the reproductive system is further supported by an increasing amount of data [18]. EVs have been seen to be involved in dynamic cell-to-cell communication through the transport of bioactive molecules related to mitochondrial functions and OS. Disrupted signaling pathways have been found to be more closely associated with impaired gamete quality, embryonic development, and implantation [28,41]. The purpose of this section is to offer a molecular foundation for comprehending the connection between mitochondria, OS, and EV-mediated signaling in human reproduction.

### 4.1. OS in Reproduction

ROS generation and the antioxidant defense mechanisms that combat them are out of balance, and this causes OS. ROS function as essential signaling molecules under physiological conditions but exert detrimental effects at elevated levels [66,67].

Sperm capacitation, hyperactivation, the acrosome reaction, and oocyte maturation are all impacted by ROS at physiological quantities. Tyrosine phosphorylation pathways are ROS-regulated events required for fertilization competence in sperm cells. Redox signaling is also implicated in the development of meiotic cells and the differentiation of cells in oocytes and embryos [68,69,70].

However, these compounds are damaged due to the excessive production of ROS. Sperm cells are especially susceptible to lipid peroxidation due to the high concentration of polyunsaturated fatty acids and low cytoplasmic antioxidant potential. Decreased rates of fertilization, low-quality embryos, and miscarriages are closely correlated with the oxidative damage of sperm DNA [71,72]. In the female reproductive system, excessive OS can lead to a decline in folliculogenesis, oocyte quality, and endometrial receptivity [73,74].

Aging, environmental pollutants, inflammation, and mitochondrial dysfunction are some of the causes of OS in the reproductive systems of both sexes [75]. Most importantly, endometriosis, polycystic ovary syndrome (PCOS), and unexplained cases of infertility are some of the reproductive system diseases in which OS is significant [76,77]. This emphasizes the significance of understanding the regulation of redox balance in the reproductive microenvironment and the role of intercellular communication mechanisms like EVs in the process.

### 4.2. Mitochondrial Function in Gametes and Embryos

Mitochondria are essential for an individual’s successful reproduction because they regulate cellular energy metabolism and redox balance. The maintenance of cellular viability and energy-demanding processes depends on the functionality of the mitochondria in male and female gametes [78,79].

Mitochondria are found in the midpiece of the spermatozoa and are responsible for the production of ATP using oxidative phosphorylation. Decreased motility, increased ROS production, and reduced fertilization capacity are some of the effects of mitochondrial dysfunction in sperm cells [80,81]. Furthermore, among the variables that are impacted in male infertility are ATP synthesis and mitochondrial membrane potential [80].

The mitochondria are the major source of ATP required for chromosomal segregation, formation of meiotic spindles, and cytoplasmic maturation of oocytes. The quality of oocytes is largely influenced by the number, distribution, and functional competency of mitochondria. Decreased fertility and poor outcomes of ART are directly linked to decreased mitochondrial function with age, including a reduction in ATP production and the acquisition of mtDNA mutations [17,82].

The activity of mitochondria is dynamically regulated in the early stages of embryonic development. Division, differentiation, and metabolic regulation are all dependent on the mitochondria that are initially present in the embryo. Mitochondrial activity disruptions may be the cause of implantation failure and embryonic arrest [83,84].

Regulation of the activity of mitochondria and the redox balance is crucial because mitochondria are involved in the production of ATP and ROS. New research reveals the role that EVs play in this regulation through the mobility of ROS and mitochondria [85,86].

### 4.3. EV Cargo in Redox Signaling

These bioactive substances have both direct and indirect effects on redox homeostasis. Antioxidant enzymes are essential in the detoxification of ROS and include glutathione peroxidase (GPx), catalase, and superoxide dismutase (SOD). By delivering antioxidant enzymes to recipient cells, EVs can enhance cellular antioxidant capacity and protect against ROS-induced damage [87].

Antioxidant compounds carried by EVs are believed to play a role in the enhancement of sperm viability, maturation of oocytes, and embryo development during reproduction [41,42]. Moreover, EVs contain non-coding RNAs such as microRNAs (miRNAs), which control genes associated with OS reactions [88].

Given that miRNAs are associated with mitochondrial processes and regulate inflammation and apoptosis, they may also play a role in maintaining redox homeostasis. MiRNA expression in infertile patients has been shown to vary and is believed to play a role in the regulation of OS responses [89,90].

Particularly in reproductive biology, the processes controlling selective cargo loading onto EVs are still poorly understood [30,33]. According to available data, the selective incorporation of mitochondrial proteins, mtDNA fragments, lipids, and redox-regulatory RNAs into EVs may be influenced by oxidative stress, mitochondrial malfunction, intracellular calcium signaling, and endosomal sorting complexes [30,33,85,86]. Furthermore, cargo selection and vesicle heterogeneity seem to be influenced by RNA-binding proteins and tetraspanin-associated processes [30,36]. Endocytosis, membrane fusion, receptor-mediated internalization, and phagocytosis are some of the ways that recipient cells might absorb EVs once they are released [36]. Nevertheless, little is known about how these pathways contribute differently to reproductive tissues. Crucially, there is currently a lack of direct causal evidence demonstrating functional mitochondrial transfer and metabolic reprogramming in recipient reproductive cells, and many studies describing EV-mediated redox and mitochondrial signaling remain largely associative [84,85,86].

In addition, EVs may contain oxidized phospholipids, lipid peroxidation-associated metabolites, and redox-active lipids capable of modulating oxidative stress pathways in recipient cells. Notably, environmental circumstances play a crucial role in EV-mediated redox signaling. Although EVs play a critical role in maintaining redox homeostasis in a physiological environment, they might also facilitate the spreading of OS signaling in a pathological environment, leading to cellular dysfunction [18,91].

The complexity of EV-mediated communication and its importance in reproductive health and condition are emphasized by this dual role of EVs in redox signaling. The main components of EV cargo involved in redox and mitochondrial signaling and their functional roles in reproduction are summarized in Table 2.

### 4.4. EV-Mediated Mitochondrial Transfer

One of the most intriguing features of EV biology may be their ability to facilitate the intercellular transport of mitochondrial components. It is clear that EVs can move proteins, DNA, and even mitochondria, and this could be a way for metabolic and bioenergetic information to be transferred between cells [86,96].

This has been proposed as a potential mechanism for partially restoring cellular bioenergetics in dysfunctional cells. Experimental studies suggest that EV-mediated transfer of mitochondrial components may influence ATP production, oxidative stress responses, and mitochondrial activity in recipient cells, although the functional integration and long-term stability of transferred material remain incompletely understood [97].

Preclinical evidence suggests that oocytes may receive mitochondrial-associated components through EVs derived from surrounding somatic follicular cells, hence increasing the metabolic potential of the oocyte. Similarly, EV-mediated mitochondrial signaling has been hypothesized to influence sperm function and oxidative stress resistance [17,98].

However, the transfer of mitochondria via EVs may potentially have adverse effects, particularly if faulty mitochondrial components or mutant mtDNA are involved. Therefore, it is anticipated that the outcome of the transfer of mitochondria by EVs would depend on the physiological state of the donor cells and the integrity of the components of mitochondria [99,100].

The mechanisms that govern the inclusion of mitochondria into EVs, the reception and integration into target cells, and the functional integration are still poorly understood, although this area is gaining increasing attention [101]. To better understand these mechanisms and to determine the importance of these processes, further studies are required.

Current research is investigating whether mitochondrial material delivered by EVs remains bioenergetically functional following uptake by recipient reproductive cells [86,96,100]. To distinguish actual functional mitochondrial transfer from passive transfer of mitochondrial fragments or stress-associated cargo, further mechanistic and long-term research is needed [91,95,101].

## 5. Multi-Omics Profiling of EV Cargo

EVs provide valuable biological information in human reproduction, as their molecular complexity reflects the dynamic physiological state of the cells of origin. The multi-molecular characterization of EV cargo is now feasible thanks to recent developments in high-throughput omics technologies. These technologies enable the identification of complex regulatory molecules in EVs that regulate essential reproductive functions [42,102].

Multi-omics approaches provide a comprehensive understanding of complex regulatory networks and associated pathways, whereas single-omics approaches offer a limited and fragmented view of EV biology. Multi-omics technologies have the potential to offer significant benefits in the field of reproductive medicine. Both the processes of human reproduction and the trustworthy biomarkers of human reproduction may be revealed by these technologies [103,104].

### 5.1. Transcriptomics

In transcriptomic studies, EV RNA content, such as mRNAs, miRNAs, long non-coding RNAs (lncRNAs), and circular RNAs (circRNAs), has been the main focus. Given their stability in EVs and their capacity to affect target cell gene expression via a post-transcriptional mechanism, miRNAs are the most researched of these [105,106].

The regulation of reproductive processes, including spermatogenesis, oocyte maturation, fertilization, and embryo development, is largely dependent on EV-mediated miRNAs [107]. For instance, a set of miRNAs present in seminal plasma EVs has been associated with male infertility and sperm motility, while those present in FF EVs are associated with embryo quality and oocyte competence. These non-coding RNAs play a critical role in modulating reproductive functions through pathways such as OS, apoptosis, and mitochondrial activity [47,108].

In addition to miRNAs, EVs carry mRNAs that can be translated into proteins in recipient cells, along with lncRNAs and circRNAs involved in gene regulation. All these types of RNA play important roles in modulating the cell’s response in the reproductive microenvironment [109]. Notably, the transcriptome patterns in the EVs have changed in pathological conditions such as endometriosis, PCOS, and infertility; the processes underlying these changes are unknown, which makes them prospective markers for diagnosis and prognosis [110,111].

Importantly, emerging integrative analyses suggest that EV-derived miRNA and lncRNA signatures may correlate with proteomic and metabolomic alterations associated with mitochondrial metabolism, OS regulation, and embryo developmental competence, highlighting the importance of cross-layered multi-omics approaches in reproductive biomarker discovery [19,20,21,90,103,104].

### 5.2. Proteomics

The functional chemical components responsible for intercellular communication can be readily elucidated through proteomic analysis of EVs. Membrane receptor proteins, signaling proteins, enzymes, and structural proteins are among the proteins linked to EVs; these proteins represent the physiological condition and biological origin of the cell source. Various biological events in reproductive biology, including sperm maturation, capacitation, fertilization, and embryo-maternal interactions, have been associated with proteins secreted in EVs [112,113]. For example, proteins secreted in endometrial EVs, including adhesion proteins and cytokines, play a role in embryo implantation, while proteins secreted in epididymal EVs, including proteins required for sperm motility, have been associated with epididymal function [42,114].

In further support of their role in redox–mitochondrial signaling, proteomic studies have identified proteins associated with inflammation, OS, and mitochondrial function in EVs. ART and reproductive diseases have been associated with differential expression of proteins in EVs, indicating their use as biomarkers. For instance, decreased expression of heat shock proteins, antioxidant enzymes, and metabolic regulators in EVs has been linked to decreased fertility [112,115].

Integrating quantitative proteomics with other omics layers can provide a deeper understanding of EV-mediated signaling networks and enhance the ability to link protein expression levels to upstream regulatory mechanisms and downstream functional effects [116].

### 5.3. Metabolomics

The detailed analysis of small molecules and metabolites in biological systems is the primary goal of metabolomics. The EV-associated metabolites are a signal of the parent cell’s metabolic status and can influence the recipient cell’s metabolic and redox state [117].

Cellular homeostasis, OS, and signals related to energy metabolism in the setting of reproduction were found in the metabolome study of EVs. Amino acids, lipids, ROS, and important metabolites derived from major metabolic pathways, including glycolysis and the tricarboxylic acid cycle, are important metabolites found in EVs [112,118].

As lipids are integral components of the EV membrane, playing a role in the formation, stability, and signaling of EVs, lipidomics, a sub-discipline of metabolomics, is particularly relevant to the study of EVs. Changes in lipid composition, including phospholipids, sphingolipids, and cholesterol, can affect EV membranes, affecting cell membrane fluidity and EV interaction with the target cell. Moreover, lipid peroxidation products in EVs are biomarkers of OS, which can lead to cell dysfunction in the reproductive system [119,120].

Metabolic markers associated with the quality of oocytes, sperm, and embryos have been identified using metabolomics analysis of EVs in FF, seminal plasma, and culture medium from ART procedures. This indicates the potential of EV metabolites as biomarkers in ART procedures, which are minimally invasive, and as markers of reproductive potential [121].

### 5.4. Microbiome-Derived EVs

Through its impact on tissue homeostasis, inflammation, and immunological responses, the reproductive tract microbiome plays a critical role in regulating reproductive health. In addition to cell-to-cell contact, microorganisms produce EVs known as outer membrane vesicles (OMVs) in bacteria [122].

The components found in microbiome-derived EVs are varied and may include proteins, lipopolysaccharides, nucleic acids, and metabolites, which may interact with host cells and affect reproductive health. Microbial EVs may play a role in the regulation of immunological tolerance, endometrial receptivity, and protection against pathogens in the female reproductive system [41]. Conversely, endometriosis, PID, and implantation failure are associated with microbiome dysbiosis and the production of pathogenic EVs, which may contribute to inflammation [123].

The recently discovered evidence that microbial EVs could alter the host cell’s mitochondrial activity and redox status provides more proof of the connection between the microbiome and EV-mediated signal transduction. For instance, bacterial EVs may induce OS or alter antioxidant balance, potentially disrupting cellular homeostasis in reproductive contexts [18,124].

Nevertheless, distinguishing host-derived EVs from microbiome-derived vesicles in heterogeneous reproductive samples such as vaginal, cervical, or uterine fluids remains technically challenging. Overlapping vesicle size distributions, shared membrane characteristics, and the absence of universally validated microbial EV-specific markers complicate accurate characterization and isolation. In addition, contamination during sample preparation and the coexistence of host and microbial vesicles within inflammatory microenvironments may introduce significant analytical bias. Advanced approaches, including single-EV profiling, high-resolution flow cytometry, multi-omics integration, and microbial membrane-specific marker analysis, may improve discrimination between host- and microbiome-derived EV populations in future studies.

In reproductive science, the integration of host cell-derived EV multi-omics profiles and microbiome is an exciting new frontier. The methods could uncover new host-microbe interactions and find indicators that show how the host and microbiome affect fertility and reproductive success [125]. The main multi-omics approaches used to characterize EV cargo and their relevance in reproductive biology are summarized in Table 3.

Despite the substantial insights provided by individual omics approaches, EV biology in human reproduction is increasingly recognized as a systems-level network involving dynamic interactions among transcriptomic, proteomic, metabolomic, lipidomic, and microbiome-derived signals [19,20,21]. Integrative multi-omics analyses may reveal mechanistic relationships that cannot be identified through isolated datasets alone [19,20,21,22]. For example, EV-derived miRNAs regulating mitochondrial biogenesis, OS responses, and apoptosis may correlate with proteomic alterations involved in mitochondrial metabolism and redox homeostasis [90]. Similarly, metabolomic and lipidomic signatures may reflect downstream functional consequences of transcriptomic and proteomic remodeling within recipient reproductive cells [117,119,120]. Emerging computational frameworks integrating EV transcriptomics, proteomics, metabolomics, and microbiome-associated datasets with AI-driven network analyses have demonstrated increasing potential for identifying interconnected molecular pathways and predictive biomarker signatures relevant to embryo competence, endometrial receptivity, and ART outcomes [22,103,104,124]. Nevertheless, true cross-layered validation studies in reproductive EV biology remain limited, and further systems-level mechanistic investigations are necessary to improve biological interpretation and clinical translation [19,20,21,22].

## 6. AI and ML in EV-Based Biomarker Discovery

Large-scale, multi-dimensional datasets that can capture the chemical complexity of EVs may now be produced thanks to the quick development of HT techniques. Although these datasets have strong potential in reproductive medicine, their high dimensionality and non-linear interactions among variables complicate their analysis [126]. AI and ML are recognized as essential tools for identifying predictive biomarkers and advancing precision medicine in human reproduction by extracting meaningful patterns from complex datasets [127].

The AI approach of integrating multi-omics datasets can prove to be a highly effective strategy in the field of EVs to establish the link between molecular markers and clinical outcomes. ML techniques can identify latent relationships among variables in integrated transcriptomic, proteomic, metabolomic, and microbiome datasets that are not detectable using conventional statistical methods. This is not possible with traditional statistical techniques. These techniques can be quite effective in predicting patient reproductive failure, embryo selection, and ART outcomes [128,129].

### 6.1. Why AI Is Needed

The inherent difficulties associated with reproductive biology and “omics” studies are also one of the driving factors for employing AI for identifying biomarkers through the use of EV-based methods. The first is that, as described, the datasets for EV studies are high-dimensional, with thousands of variables measured for a relatively small number of subjects. This may contribute to “overfitting,” or the potential for traditional statistical analysis to fail [130,131].

The second is that, as described, biological systems are complex and nonlinear, involving multiple systems and pathways. For example, systems such as OS, mitochondrial function, immunologic control, and hormonal regulation are all interconnected and play a role in reproductive outcomes [132]. While ML is designed to analyze such complexity, traditional statistical analysis may fail to capture these complex interrelations [133].

Third, additional levels of heterogeneity are introduced depending on differences in EV isolation approaches, sample types, and patient populations. Identification of invariant patterns through AI approaches can help mitigate this problem [134].

Ultimately, developing a framework to integrate diverse multi-omics data into a unified analytical model is essential. AI supports this by applying dimensionality reduction, feature selection, and data integration to derive physiologically relevant insights from complex datasets [128].

### 6.2. ML Approaches

Several methods involving machine learning have been applied in the field of EV-based biomarker research, each with distinct advantages dependent on the type of data and the study’s goals [131].

Predictive modeling in machine learning commonly employs techniques such as support vector machines, random forests, gradient boosting machines, and logistic regression. Such algorithms enable the identification of EV-based markers associated with clinical outcomes, including implantation success and pregnancy [135,136].

To identify patterns in data, several machine learning algorithms can be used, including principal component analysis, k-means clustering, and hierarchical clustering. Such algorithms can provide insights into the biological heterogeneity of disease processes, particularly through the identification of patient groups associated with specific reproductive traits [83,137].

Deep learning, an artificial intelligence technique based on artificial neural networks, is increasingly used due to its ability to model highly complex, non-linear relationships. Convolutional neural networks and recurrent neural networks have been tested for biomedical data [138,139]. Although deep learning has significant potential to enhance prediction accuracy and handle multimodal data, it remains underutilized in EV research [131].

Moreover, feature selection techniques such as recursive feature elimination and LASSO regression are critical for identifying relevant features while maintaining model simplicity. Network-based approaches and systems biology help enhance the interpretability of the model by establishing molecular connections [140,141].

### 6.3. Applications in ART

The use of AI and ML techniques in assisted reproductive technologies has grown, as has interest in using data from EVs for clinical decision-making [131,142].

Recent studies have begun integrating AI/ML approaches with EV-associated molecular data to improve reproductive outcome prediction [22,127,128,129,130,131]. For example, ML models combining metabolomic and embryologic datasets have demonstrated improved prediction of embryo implantation potential, with reported AUC values exceeding 0.80 in selected cohorts [143]. Similarly, transcriptomic analysis of uterine fluid EVs combined with Bayesian and systems biology approaches has shown promise for predicting endometrial receptivity and pregnancy outcomes [103]. Several studies have also utilized FF proteomics and metabolomics datasets to identify biomarkers associated with embryo quality, fertilization success, and implantation rates [121].

Despite these promising findings, most currently available AI-assisted reproductive biomarker studies remain limited by relatively small cohort sizes, lack of external multicenter validation, methodological heterogeneity, and variability in EV isolation and preprocessing workflows [22,129,130,131,141,142]. Consequently, many proposed predictive models remain exploratory and have not yet achieved routine clinical implementation [3,4,141].

Predicting the quality of embryos and their implantation potential is one of the most promising uses. ML models can detect biomarkers linked to embryo viability and developmental competency by including EV-associated molecular signatures from FF, embryo culture media, or endometrial secretions. This could lessen the need for invasive procedures and enhance embryo selection techniques [121,143,144,145].

Predicting ART results, such as fertilization success, implantation rates, and live birth rates, is another important application. When paired with clinical factors, machine learning models built on multi-omics EV data can produce individualized predictions that inform treatment choices and enhance patient care [143,146].

AI-powered methods for evaluating endometrial receptivity are also being investigated. The uterine environment’s preparedness for implantation is reflected in molecular signals seen in endometrial-derived EVs. Implantation success rates may increase, and embryo transfer timing may be improved by incorporating these signals into predictive models [23,147].

EV-based indicators found by ML analyses may help assess sperm quality in male infertility beyond traditional semen analysis, allowing for more accurate diagnosis and focused treatment approaches [148,149].

Collectively, these applications highlight the potential of AI-integrated EV research to advance reproductive care through non-invasive, data-driven, and personalized strategies for fertility evaluation and therapy.

### 6.4. Methodological Challenges and Translational Barriers

Although both AI and ML technologies hold great potential for creating EV-based biomarkers, methodological and computational challenges still need to be addressed [22,150].

One major limitation in developing reliable biomarkers has been access to large-scale, high-quality data sets. The high-dimensional nature of multi-omics data has meant that many studies in reproductive health suffer from limited sample size, which in turn creates the possibility of “overfitting” in ML algorithms. The limited sample size has been a major limitation, especially in developing reproducible biomarker signatures [21,126,141].

Despite promising developments, many AI/ML-based biomarker studies in reproductive medicine remain limited by small cohort sizes, insufficient external validation, and high risk of overfitting [127,128,129,130,131,150]. The high dimensionality of EV-derived multi-omics datasets, combined with relatively low sample numbers, increases the likelihood that predictive models capture dataset-specific noise rather than biologically robust signatures [21,22,126]. Reproducibility remains severely compromised by batch effects caused by differences in sample collection, EV isolation protocols, sequencing platforms, and data preparation techniques [105,134]. Additionally, many currently proposed classifiers lack prospective clinical testing and independent multicenter validation, which limits their translational usefulness [141,150]. Therefore, rigorous validation frameworks, transparent reporting standards, harmonized preprocessing pipelines, and XAI approaches will be essential before EV-based AI models can be reliably implemented in clinical reproductive medicine [22,141,150].

Heterogeneity in data structure, size, and noise across transcriptomic, proteomic, and metabolomic datasets poses a significant challenge for multi-omics integration, necessitating computational frameworks that can handle complex, high-dimensional data. Finding patterns in data sets that are physiologically relevant has been made possible by the creation of trustworthy integration techniques [22,105].

Another significant problem with the practical application of the model is the lack of model interpretability. The “black-box” nature of complex algorithms, especially in deep learning, is a significant drawback, particularly when high performance comes at the expense of interpretability. The use of XAI is crucial for understanding the importance of features and underlying biological processes, as the lack of model interpretability remains a significant limitation [141,150].

In reproductive medicine and ART, model interpretability is particularly important because clinical decisions directly influence embryo selection, implantation strategies, and patient counseling [3,4,150]. XAI approaches may improve clinician trust by identifying the molecular features, pathways, and EV-associated biomarkers contributing most strongly to model predictions [22,141]. Techniques such as feature importance ranking, SHAP (Shapley Additive Explanations) analysis, and attention-based modeling may help clinicians better understand AI-driven recommendations and evaluate their biological plausibility [127,141]. Furthermore, interpretable AI frameworks may facilitate regulatory approval, improve transparency, and support the integration of AI-assisted decision-making into routine reproductive clinical practice [22,141,150].

The lack of external validation and model generalizability is a major limitation, particularly when models are developed and evaluated using a single dataset. Cross-validation is therefore essential to ensure robust performance across different populations [136,150].

Lastly, to avoid biases and ensure clinical relevance, it is essential to carefully design and test the models. Misleading results may arise from inadequate handling of issues such as class imbalance, data leakage, and improper performance evaluation. The implementation of AI-based methods for biomarker discovery requires rigorous validation techniques, including cross-validation and prospective evaluation [141,150]. The main AI and machine learning approaches applied in EV-based biomarker discovery and their relevance to reproductive medicine are summarized in Table 4. Representative studies applying AI/ML approaches in reproductive biomarker discovery, including cohort characteristics, biological sources, algorithms, and clinical endpoints, are summarized in Table 5.

## 7. Clinical Applications

EVs are considered promising candidates for application in reproductive medicine because they have specific biological properties, stability in biological fluids, and the ability to mirror the physiological state of the cells from which they originate [155]. EV-based methods have gained increasing attention in recent years, particularly due to their potential as therapeutic agents and for diagnosis and prognosis. The integration of multi-omics and AI enhances the translational value of EV biology, enabling the development of individualized, non-invasive approaches for infertility management and ART optimization [156].

### 7.1. Diagnostic Biomarkers

The use of EVs as non-invasive diagnostic biomarkers is one of the most promising uses of EVs in the clinic. Many accessible bodily fluids, including seminal plasma, FF, uterine secretions, blood plasma, and embryo culture medium, can be employed in the collection of EVs [157]. The molecules within the EVs can provide valuable information about the possible causes of infertility, as they provide an overview of the physiological and pathological state of the reproductive organs [51].

The possible uses of EVs in the evaluation of the quality of sperm, as well as the possible causes of infertility in male reproductive organs, have been researched [41]. For instance, the possible causes of asthenozoospermia and oligozoospermia, which are forms of male infertility, have been linked to the EVs’ miRNA, protein, and OS markers. By providing molecular information about sperm, these approaches can improve or even surpass conventional semen analysis [158].

The competence of the oocyte and health of the follicle have been related to the presence of EVs in the FF [50]. Proteins and miRNAs derived from these vesicles have been considered potential markers for the developmental capacity of the oocyte, thus facilitating a detailed evaluation of the reproductive status [10]. Similarly, vesicles derived from uterine fluid or endometrial tissue may indicate receptivity and help determine the optimal time for implantation [103,154].

Most significantly, the use of EV-based diagnostics is likely to minimize patient stress and allow for longitudinal sampling through the provision of a less invasive alternative to conventional methods [159]. The potential for improved diagnostic accuracy and the provision of personalized reproductive health through the use of EV-based biomarkers, coupled with the application of multi-omics and AI, is evident [160].

### 7.2. Prognostic Biomarkers

Apart from diagnosis, EVs hold promise as prognostic biomarkers for predictive purposes, especially when ART is considered. One major gap in the practice of reproductive medicine is the need to make predictive prognoses regarding the success or failure of treatment, viability, and implantability [41].

EV-derived molecular signatures identified in FF, embryo culture medium, and uterine secretions have been correlated with key ART outcomes, including fertilization, embryo quality, and live birth rates [10,42]. For instance, miRNAs and proteins in EVs have been associated with successful pregnancy and embryos with high developmental potential, whereas others are linked to poor developmental outcomes and decreased pregnancy rates [161].

The predictive value of EV-based biomarkers can be further improved through the integration of these biomarkers with AI and ML algorithms. The ML algorithm can identify predictive patterns associated with clinical outcomes by analyzing complex data [162,163].

EV-based biomarkers offer a novel, non-invasive alternative to traditional embryo selection methods, including morphological analysis and preimplantation genetic testing. The analysis of EVs in the embryo culture medium can improve the success rates and minimize the risks of invasive procedures, particularly those involving the selection of the most implantable embryos [164,165].

In the same context, the use of endometrial receptivity biomarkers, which are derived from EVs, can improve the window of embryo transfer, thereby increasing the chances of implantation success. In conclusion, the use of EVs as predictive biomarkers can improve patient outcomes and ART-related decisions [53,154].

### 7.3. Therapeutic Potential

Besides diagnostic and predictive purposes, EVs have been considered for therapeutic roles in reproductive medicine. Their intrinsic role in intercellular communication and their capacity for carrying bioactive molecules make them good candidates for therapeutic purposes [12,155].

One of the possible roles of EV-based therapy is in modulating OS and mitochondrial dysfunction, two major contributors to reproductive failures [155].

For example, EVs carrying antioxidant enzymes, regulatory RNAs, or even mitochondrial components may have therapeutic value in maintaining normal reproductive tissues and gametes [166]. EVs derived from mesenchymal stem cells exhibit anti-inflammatory and antioxidant properties and hold therapeutic potential for conditions such as endometriosis, ovarian aging, and male infertility [167].

Regenerative medicine approaches to reproductive health may also involve the use of EVs. The use of EVs may be significant in the treatment of endometrial dysfunction or premature ovarian insufficiency, among other reproductive health concerns [168,169]. Preclinical studies have shown that EVs can promote angiogenesis, healing, and cell proliferation [170].

EVs may be further optimized to enhance their effectiveness in the treatment and prevention of reproductive health conditions. Improved approaches to loading EVs with different molecules, such as proteins, RNA, and drugs, have opened up new avenues for the use of targeted therapy. Such approaches may reduce systemic side effects by enabling targeted drug delivery to the reproductive system [171,172].

Although EV-based treatments show significant potential, several challenges must be addressed prior to their clinical implementation. These issues include questions about the safety, standardization, mass production, and approval of these treatments [155,173]. Moreover, a deeper understanding of the biodistribution, targeting, and long-term effects of EVs is necessary [174].

In addition, the potential immunogenicity and off-target effects of engineered or modified EVs remain insufficiently characterized [12,171,173,174]. Regulatory approval is further complicated by the biological heterogeneity of EV preparations, lack of universally standardized manufacturing protocols, and variability in cargo composition between studies [24,40,155,171]. Similarly, although numerous EV-associated biomarkers have shown diagnostic and prognostic promise, clinically validated threshold values and standardized cut-off ranges remain largely unavailable, limiting their routine implementation in reproductive medicine [24,155,159].

## 8. Challenges and Limitations

EV-based applications in reproductive health face biological, methodological, translational, and computational challenges, including those related to AI and ML [152].

The substantial methodological diversity introduced by various EV isolation and purification procedures is a significant obstacle in EV research [29,37,134]. Partially overlapping but physiologically separate EV subpopulations are frequently isolated using widely used techniques such as ultracentrifugation, size-exclusion chromatography (SEC), polymer-based precipitation kits, immunoaffinity techniques, and newly developed microfluidic platforms [29,37]. Variations in isolation techniques can significantly impact vesicle purity, yield, cargo composition, and subsequent multi-omics analysis [105,151]. For instance, protein aggregates and lipoproteins may be co-isolated using precipitation-based techniques, although vesicle aggregation or structural damage may result from ultracentrifugation [29,134]. Likewise, the SEC increases purity but can decrease EV recovery [37]. These methodological inconsistencies complicate cross-study comparisons and may contribute to poor reproducibility of proposed EV biomarkers [38,39,151]. Significant analytical bias is introduced into AI/ML-based biomarker discovery processes since omics datasets produced by various EV separation methods may not be directly comparable [105,126].

The absence of standardized methods for the isolation, purification, and characterization of EVs remains a major challenge. Various methods, including size-exclusion chromatography, ultracentrifugation, precipitation, and emerging approaches such as microfluidics, are employed [151]. However, results vary significantly. Such methodological differences may affect outcomes and complicate comparisons across studies. The analysis may also become complicated because of the presence of other non-EV particles, such as protein aggregates and lipoproteins [175,176].

However, the study and clinical use of the EV population are further complicated by the inherent heterogeneity of the vesicles. This is because the vesicles vary in size and characteristics even within the same bio-sample. Moreover, vesicles vary in their cellular origin and cargo [9,176]. While, the biological functions of these vesicles and the identification of specific biomarker sets remain difficult due to limitations in analytical tools that distinguish between vesicle types [177].

Significant variation is also introduced in EV research studies through pre-analytical factors. For instance, EV integrity and cargo composition are highly sensitive to sample collection methods, processing time, storage conditions, and freeze–thaw cycles [178,179]. Hormonal variation, menstrual cycle phases, ovarian stimulation protocols, and patient-specific factors, including age, metabolic status, and environmental conditions, also contribute to variability in reproductive research [180,181].

The complex and context-dependent nature of EV content presents additional challenges. The physiological state of the donor cells, as well as the environment, including OS, inflammation, and metabolism, are reflected in EV composition. Distinguishing between associative markers and causal effects remains challenging, particularly in clinically heterogeneous populations [112,182].

Furthermore, the practical application of EV-based diagnostics and treatments is hindered by translational barriers. While several potential biomarkers have been discovered, only a limited number have been thoroughly validated on large, unbiased populations [183]. The practical application of EV-based testing is also hindered by the lack of established clinical pipelines, including validated cut-offs and ranges. Prior to clinical application, challenges related to large-scale production, batch-to-batch consistency, stability, and delivery efficiency must be addressed [131].

Another major limitation is the limited understanding of the biodistribution, targeting specificity, and functional uptake of EVs in vivo [184]. Although it is known that EVs play an important role in intercellular communication, little is known about the mechanisms governing the cellular uptake of EVs and tissue-specific targeting in the reproductive system. This is affecting the prediction of the effects of EV-based therapeutic approaches [185].

Lastly, the issue of safety and regulation is another major constraint to the translation of EV-based therapy. From a regulatory perspective, the classification of EV-based therapies, particularly modified vesicles, remains evolving [186]. Evaluating the safety profile is essential before the clinical translation and application of EV-based therapies in reproductive medicine, given the potential immunogenic, off-target, and long-term effects [108].

In conclusion, EV research holds significant potential, but several challenges remain. Addressing these will require collaborative efforts to standardize, optimize, and validate EV-based therapeutic applications [155].

## 9. Future Perspectives

The integration of AI, multi-omics, and EV biology is also expected to bring significant advances to the field of reproductive medicine. Future studies are also expected to increasingly focus on utilizing the increasing understanding of EV-mediated communication to develop tools for predictive, preventative, and individualized approaches to reproductive management [20].

One of the most promising approaches is the development of integrated multi-omics frameworks that can generate a complete molecular profile of EV cargo using transcriptomics, proteomics, metabolomics, and microbiome-derived data [126]. Integrative approaches will enable the development of reliable biomarker signatures that capture the complex interactions among redox, mitochondrial, immune, and metabolic pathways [187]. Advanced computational techniques can improve the accuracy of reproductive outcome prediction by identifying clinically significant patterns in high-dimensional data [188].

It is believed that new technology will greatly enhance the sensitivity and resolution of EV analysis. Recent advances in single-vesicle analysis, sequencing, and imaging offer unprecedented opportunities to characterize EV diversity [189]. Emerging technologies can overcome key limitations of bulk analysis approaches and facilitate the identification of specific roles of functionally distinct EV subpopulations in reproductive processes [176].

The use of EV-based biomarkers in the clinic is another area that will experience further advancement in the future. To ensure the reproducibility of EV-based biomarkers, standardized approaches in the isolation, characterization, and analysis of EVs will need to be established in the future [190]. The validation of potential biomarkers requires extensive research, including longitudinal cohort studies. In the future, EV-based tests may be integrated into clinical practice to non-invasively assess endometrial receptivity, gamete quality, and embryo viability [191].

Additionally, integrating EV research with AI-based decision support systems will advance precision medicine in reproductive medicine. These systems will have the potential to provide real-time, patient-specific predictions of the outcomes of ART procedures and guide treatment accordingly. In this regard, to bridge the gap between computational advances and practical applications, explainable models will have to be developed [150].

From a therapeutic standpoint, EVs represent a promising approach for developing targeted therapies to restore reproductive function. Modified EVs could be explored as delivery vehicles for bioactive molecules, including proteins, metabolic regulators, and small RNAs [155]. Such approaches could enhance mitochondrial function, regulate OS, and promote tissue regeneration in the reproductive system. However, this would require a deeper understanding of EV biodistribution and long-term effects [18].

Moreover, integrating EV biology with emerging disciplines such as systems biology and digital health may further advance reproductive science [192]. The emergence of “digital twins” in reproductive health may result from predictive models that incorporate lifestyle, environmental, and patient data. This would enable optimization of treatment approaches through simulation [193].

Despite these advances, overcoming challenges in standardization, scalability, and regulatory approval is critical for the clinical integration of EV-based techniques. Further development in this area will require an interdisciplinary approach among engineers, biologists, bioinformaticians, and clinicians [194,195].

## 10. Conclusions

EVs are involved in intercellular communication in human reproduction, affecting the maturation of gametes, fertilization, development, and implantation. EVs contribute to redox and mitochondrial regulation by transporting proteins, lipids, nucleic acids, and mitochondrial components. These properties position EVs as dynamic mediators linking cellular signaling pathways with reproductive function and dysfunction.

The use of multi-omics approaches facilitates the analysis of EVs and the complex patterns of cargo that are present in reproductive health and disease. When combined with AI and machine learning methodologies, these technologies may improve biomarker discovery, predictive modeling, and clinical decision-making in ART. Such integrative approach facilitates the development of non-invasive and individualized approaches to fertility evaluation and treatment.

Nevertheless, several important challenges remain, including EV heterogeneity, methodological variability, limited reproducibility, and the need for rigorous clinical validation and standardization. Further interdisciplinary and mechanistic studies are required to facilitate the clinical translation of EV-based technologies. Importantly, this review proposes an integrated framework linking EV-mediated communication with redox regulation, mitochondrial signaling, multi-omics profiling, and AI-assisted biomarker discovery, thereby providing a broader conceptual perspective for the future development of precision reproductive medicine.

## Figures and Tables

**Table 1:** EVs from reproductive tissues regulate gamete maturation, fertilization, embryo development, and implantation through their molecular cargo, with implications for redox signaling, immune modulation, and biomarker development in ART.

Reproductive System	EV Source	Key Cargo	Biological Functions	Clinical Relevance
Male [46,57]	Epididymosomes (epididymal epithelium)	Proteins, lipids, miRNAs, regulatory RNAs	Sperm maturation, acquisition of motility, membrane remodeling, metabolic activation	Biomarkers of sperm quality and maturation status
Male [47,58]	Seminal plasma EVs (prostate, seminal vesicles, accessory glands)	Antioxidant enzymes, proteins, RNAs	Sperm capacitation, motility, protection against OS, redox regulation	Non-invasive biomarkers for male infertility (e.g., asthenozoospermia, oligozoospermia)
Male → Female interaction [48,59]	Seminal EVs in female reproductive tract	Immunomodulatory proteins, signaling molecules	Induction of immune tolerance to paternal antigens, support of embryo development	Potential targets for improving implantation success
Female [28,60]	FF EVs (granulosa, theca cells)	miRNAs, proteins, metabolites	Oocyte maturation, folliculogenesis, oocyte–somatic cell communication	Biomarkers of oocyte competence and embryo quality
Female [61,62]	Oviductal EVs	Proteins, RNAs, signaling molecules	Regulation of sperm capacitation, acrosome reaction, fertilization, early embryo development	Potential targets for improving fertilization outcomes
Female [54,63]	Endometrial EVs	Cytokines, miRNAs, adhesion molecules	Embryo–maternal communication, endometrial receptivity, trophoblast invasion	Biomarkers for implantation success and ART outcomes
Female (microenvironment) [64,65]	Microbiome-derived EVs/immune-related EVs	Microbial components, inflammatory mediators	Immune modulation, maintenance of reproductive homeostasis	Implicated in endometriosis, implantation failure

**Table 2:** EVs carry antioxidant enzymes, RNAs, lipids, and mitochondrial components that regulate redox balance and mitochondrial function, influencing gamete quality, embryo development, and infertility.

Cargo Type	Examples	Function	Reproductive Impact
Antioxidant enzymes [15,92]	GPx, SOD, catalase	ROS detoxification	Protect sperm, oocytes, embryos
miRNAs [90,93]	Redox-related miRNAs	Regulate OS genes	Influence gamete quality, embryo development
Lipids/metabolites [94]	Peroxidation products	Reflect oxidative status	Biomarkers of OS
Mitochondrial components [95]	mtDNA, proteins	Bioenergetic support	Improve or impair mitochondrial function

**Table 3:** Overview of major omics approaches used to characterize EV cargo, highlighting their functional roles, contributions to reproductive biology, and potential as non-invasive biomarkers in fertility assessment and ART.

Omics Layer	EV Cargo Analyzed	Key Functions	Biological & Clinical Insights	Biomarker Potential
Transcriptomics	miRNAs, mRNAs, lncRNAs, circRNAs	Regulation of gene expression; modulation of OS, apoptosis, and mitochondrial pathways [105,106,107,108,109]	Spermatogenesis, oocyte maturation, fertilization, embryo development [107,108]	Biomarkers of sperm quality, oocyte competence, embryo viability, infertility disorders [110,111]
Proteomics	Enzymes, receptors, cytokines, structural proteins	Cell signaling, redox regulation, immune modulation, metabolic control [112,113]	Sperm motility, capacitation, embryo–maternal communication, implantation [114]	Biomarkers for ART outcomes, implantation success, reproductive diseases [112,115,116]
Metabolomics/ Lipidomics	Amino acids, lipids, ROS-related metabolites, metabolic intermediates	Regulation of cellular metabolism, redox balance, membrane dynamics [117,118,119,120]	Oocyte quality, sperm function, embryo development [118,121]	Non-invasive biomarkers in FF, seminal plasma, and embryo culture media [121]
Microbiome- derived EVs	Proteins, lipopolysaccharides, nucleic acids, metabolites	Host–microbe communication, immune modulation, inflammation, redox signaling [122,124]	Endometrial receptivity, immune tolerance, microbiome–fertility interactions [123]	Biomarkers for endometriosis, implantation failure, and reproductive dysbiosis [123,125]

**Table 4:** ML approaches in EV-based biomarker discovery and their applications in reproductive medicine.

Category	Methods	Purpose	Applications in Reproduction	Limitations
Predictive Models [135,136]	Support vector machines, random forests, gradient boosting, logistic regression	Predict clinical outcomes and identify biomarkers	Embryo selection, implantation success, pregnancy prediction	Overfitting, limited interpretability
Unsupervised Learning [151,152]	PCA, k-means clustering, hierarchical clustering	Identify patterns and patient subgroups	Stratification of infertility phenotypes, biological heterogeneity	May lack clinical interpretability
Deep Learning [4,139]	CNNs, RNNs	Model complex, non-linear relationships	Multimodal data integration, embryo assessment	“Black-box” nature, requires large datasets
Feature Selection [140,141]	LASSO, recursive feature elimination	Identify relevant biomarkers and reduce dimensionality	Selection of EV-derived molecular signatures	Risk of information loss
Systems Biology/Network Approaches [21,152]	Network analysis, integrative modeling	Link molecular interactions and pathways	Multi-omics integration and mechanistic insights	Computational complexity
Clinical Applications [150,153]	Integrated ML pipelines	Decision support and personalized medicine	ART outcome prediction, sperm quality assessment, endometrial receptivity	Requires validation and standardization

**Table 5:** Representative AI/ML studies in reproductive biomarker discovery integrating omics or EV-associated datasets.

Study	Biological Source	Cohort Size	Omics/ Data Type	AI/ML Method	Clinical Endpoint	Key Findings
Wang et al., 2022 [135]	IVF clinical datasets	24,730 IVF/ICSI cycles	Clinical and embryologic data	Random forest, logistic regression	Clinical pregnancy prediction	Random forest outperformed logistic regression in ROC analysis
Cheredath et al., 2023 [143]	Embryo culture metabolomic and embryologic datasets	56 infertile couples undergoing single blastocyst transfer	Metabolomics and embryology	ML integration models	Embryo implantation prediction	Integration of metabolomic and embryologic data improved implantation prediction compared with conventional embryo assessment alone
Bereczki et al., 2025 [136]	IVF patient cohort	1243 IVF/ICSI cycles	Clinical reproductive variables	ML predictive models	IVF outcome prediction	ML models demonstrated strong predictive performance for IVF success and highlighted the importance of female preprocedural factors
Marzanati et al., 2025 [154]	Uterine fluid EVs	82 uterine fluid EV samples	EV transcriptomics	Bayesian modeling and systems biology approaches	Endometrial receptivity and pregnancy prediction	Transcriptomic profiling of uterine fluid EVs demonstrated potential for non-invasive prediction of endometrial receptivity and pregnancy outcomes
Przewocki et al., 2024 [144]	FF	30 patients	Proteomics	Bioinformatic and proteomic integration analyses	Embryo developmental competence prediction	FF proteomic profiling identified protein signatures associated with normal embryonic development
Toporcerová et al., 2025 [145]	Embryo secretome and embryo culture media	Narrative and experimental embryo secretome datasets	Secretome profiling	AI-assisted biomarker interpretation and computational analyses	Embryo quality assessment and IVF outcome prediction	Embryo secretome profiling demonstrated potential utility for non-invasive assessment of embryo developmental competence and IVF outcomes

## Data Availability

No new data were created or analyzed in this study.

## Abbreviations

The following abbreviations are used in this manuscript:

EVs	Extracellular Vesicles
OS	Oxidative Stress
ART	Assisted Reproductive Technologies
ROS	Reactive Oxygen Species
mRNA	Messenger RNA
miRNA	MicroRNA
ML	Machine Learning
AI	Artificial Intelligence
lncRNA	Long non-coding RNA
circRNA	Circular RNA
PCOS	Polycystic Ovary Syndrome
SEC	Size-exclusion Chromatography
